# Comprehensive Assessment of Lifetime Cigarette Smoking and Its Association with Health-Related Quality of Life among Older US Adults—A Cross-sectional Study

**DOI:** 10.21203/rs.3.rs-5319716/v1

**Published:** 2024-11-17

**Authors:** James D. Sargent, Jenny E. Ozga, Cassandra A. Stanton, Zhiqun Tang, Laura M. Paulin

**Affiliations:** Dartmouth College Geisel School of Medicine; Westat, Behavioral Health and Health Policy Practice; Westat, Behavioral Health and Health Policy Practice; Westat, Behavioral Health and Health Policy Practice; Dartmouth-Hitchcock Medical Center

**Keywords:** health-related quality of life, smoking, tobacco, cross-sectional study

## Abstract

**Background:**

Cigarette smoking is an established risk factor for lower health-related quality of life (HRQOL). Studies to date have not used comprehensive measures of tobacco exposures across the life course. We examined the association between a lifetime cigarette smoke exposure index (LCSEI) and HRQOL among older US adults.

**Methods:**

Cross-sectional analysis of N=7,001 U.S. adults ≥40y from Wave 5(2018–19) of the Population Assessment of Tobacco and Health Study. The 11-point LCSEI included heaviness of current smoking, pack-years, childhood smoking, and second-hand smoke exposure. HRQOL measures included PROMIS global physical and mental health (GPH, GMH) scores. We estimated the independent association between LCSEI scores and mean GPH or GMH separately using multivariable linear regression adjusted for sociodemographics, body mass index, geographic location, and weekly exercise.

**Results:**

Sociodemographics were reflective of the US population over 40 years; 6.7% smoked during childhood, and 16.9% currently smoked (2.1% <10cig/d, 4.8% 11–20 cig/d, and 10.0% >20 cig/d). Mean (std dev) for the LCSEI, GPH and GMH were 2.4 (2.9), 14.8 (3.1), and 14.5 (3.3) respectively, and 15% had LCSEI scores of 5 or more. In the multivariable analysis, the LCSEI retained a strong association with GPH and GMH, −0.20 (−0.23, −0.17) and −0.22 (−0.25, −0.18) respectively for each 1-point increase in the LCSEI. The LCSEI—HRQOL associations over its 11-point range (−2.14 GPH, −2.16 GMH) were significantly higher than for education (−0.44 and −0.77) and about 30% higher than for the negative influence of poverty (<25K/yr) vs. affluence (>=100K/yr) (−1.61 and −1.65). Sensitivity analysis found that associations remained significant even after further adjustment for smoking-related diseases.

**Conclusion:**

In this US sample, associations between life course cigarette smoking and both physical and mental health were as strong as associations that contrasted extremes of socioeconomic status.

## INTRODUCTION

The adverse effects of cigarette smoking on health-related quality of life (HRQOL) has been well documented over the 3 decades, since early studies on this topic was published.(1) A recent meta-analysis concluded that many aspects of cigarette smoking were associated with HRQOL, including current smoking, current smoking intensity, pack-years of smoking and second-hand smoke exposure.(2)

Measurement of smoking in HRQOL studies has not typically been comprehensive; many previous studies focused only on a single aspect of cigarette smoking exposure. For example, most studies of the association between current smoking and HRQOL compared only current smoking with former and never smoking status.(1, 3–9) One study that also assessed smoking history found that HRQOL was associated with current smoking status, heaviness of smoking, and time since quitting.(10) Similarly, studies of second-hand smoke and HRQOL have tended to focus only on nonsmokers,(11–14) despite evidence that living with a smoker affects disease symptoms independently from current smoking status.(15)

The present cross-sectional study focuses on the association between various aspects of cigarette smoking and HRQOL in the general population of older adults. We capitalize on a nationally representative US survey that measured multiple cigarette smoking outcomes reaching back to childhood to create a life course smoking index that includes heaviness of current smoking, pack-years of smoking, childhood onset of smoking < 15 years, and second-hand smoke exposure. We assess its association with global measures of physical and mental health derived from the Patient-Reported Outcomes Measurement Information System (PROMIS) in adults aged 40 years and older.

## METHODS

### Study Participants

**Figure 1:**
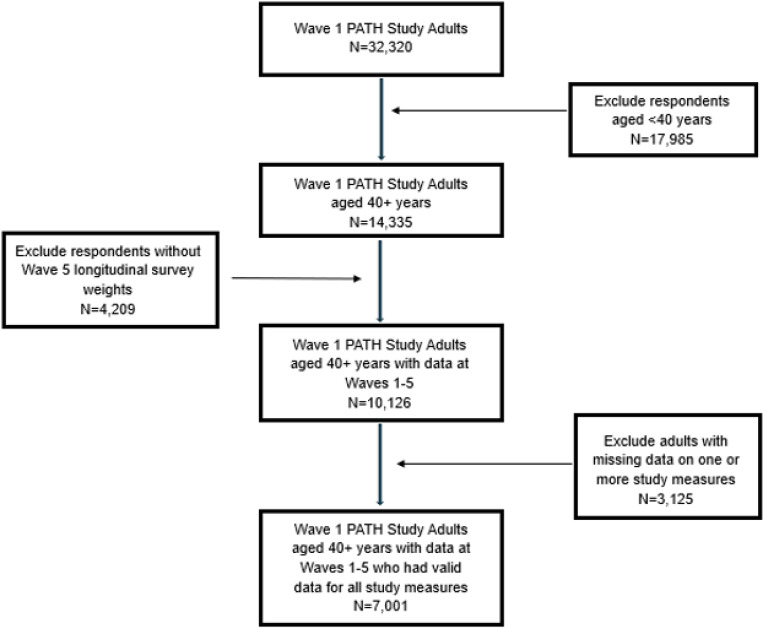
Flow diagram for study sample.

Cross-sectional data were drawn from the Population Assessment of Tobacco and Health (PATH) Study, a national cohort survey of U.S. youth and adults. Survey data were collected from adults (aged 18+) in 2018–2019 (Wave 5) in respondents’ households using computer-assisted self-interviews administered in English or Spanish as appropriate. The PATH Study questionnaire and related documentation has been published elsewhere.(16) The present study used the Wave (W) 5 Adult Restricted Use Files and was limited to the N = 14,335 adults aged 40 + years at W5, the age when many smoking-related diseases that affect HRQOL begin to manifest themselves (Fig. 1). Of these, 4,209 (22.7%) were lost to follow-up from W1 to W5; the W5 all-waves regression weights employed in our analysis accounted for oversampling at W1 and attrition between W1 and W5 in order to reduce bias and obtain estimates for the U.S. population.

### Primary Outcome PROMIS measures of global physical (GPH) and mental health (GMH)

PROMIS includes over 300 measures of physical, mental, and social health for use with the general population and with individuals living with chronic conditions.(17) The PATH Study employed the PROMIS Global-10 sub-questionnaire, which is used to measure global health. Our outcomes included a four-item global physical health (GPH) subscale and global mental health (GMH) subscale (both scored 4–20).(18) The scales have been validated through their correlation with similar domains in the more comprehensive EuroQOL-5D (EQ-5D) instrument.(19) Higher scores indicate a higher HRQOL. Among the final analytic sample, both subscales had good internal reliability, with Cronbach’s alpha of 0.73 and 0.82 for GPH and GMH, respectively.

### Main Exposure Variable

A lifetime cigarette smoke exposure index (LCSEI) was created using the following survey items. Among adults who ever smoked, current smoking was assessed by asking, “Do you NOW smoke cigarettes every day, some days, or not at all?”, recoded to never (< 100 cigarettes lifetime), former (> 100 lifetime, not at all now), and current (> 100 lifetime, every day or some days). Smoking intensity (“On average how many cigarettes do you NOW smoke per day”) was used to categorize adults who currently smoked into light ( < = 10 cigarettes/day), medium (11–20 cigarettes/day) and heavy (> 20 cigarettes/day) smoking. Index scores were 0 for never or former smoking, and 1, 2, or 3 for light current, medium current, or heavy current smoking, respectively. People who had ever smoked (at least 100 cigarettes in their entire life) were asked, “How old were you when you FIRST started smoking regularly?” Childhood smoking (age of smoking onset < 15 years) has been associated with chronic obstructive lung disease (COPD) independent of heaviness of current smoking and pack-years of smoking.(20) Therefore, index scores for age of onset were 0 for never smoking, 1 for age of onset 15 + years of age, and 2 for age of onset < 15 years of age. Based on smoking onset and current smoking intensity, pack years was determined for adults who currently smoked. Adults who formerly smoked were asked how many years since they quit smoking cigarettes and usual amount smoked, which were used to determine their duration and pack years of smoking. Index scores were 1, 2, and 3 respectively for 1–20, 21–40, and > 40 pack years of smoking among adults who ever smoked. Finally, second-hand smoke exposure was assessed based on responses to the question, “In the past 7 days, number of hours that you were in close contact with others when they were smoking?” and was given index scores of 0, 1, 2, or 3 for 0, 1–10, 11–20, and > 20 hours/week, respectively.

Each of the above measures have previously been shown to be independently associated with COPD.(20) Index scores for current smoking, smoking intensity, age of cigarette onset, pack-years of smoking, and second-hand smoke exposure were summed to create a total LCSEI score, which ranged from 0 (never smoking, no second-hand smoke exposure) to 11 (current heavy smoking, age of onset < 15 years, > 40 cigarette pack-years, and > 20 hours/week second-hand smoke exposure). The LCSEI was a reliable index (Cronbach’s alpha = 0.83).

### Covariates

Covariates included age, gender, race, annual household income, educational attainment, geographic location, body mass index, and weekly hours of moderate-intensity exercise, and were categorized as shown in Table 1. We did not adjust the association between the LCSEI and HRQOL for tobacco-related diseases, like COPD, because these diseases are on the causal pathway between cigarette smoking and impaired HRQOL.

### Statistical Analysis

We first created a limited dataset that included the GPH and GMH outcome measures, the main exposure variables (smoking status, smoking intensity, age of cigarette onset, cigarette pack-years, and second-hand smoke exposure), and covariates (age, gender, race, income, education, geographic location, exercise, and BMI). In this limited dataset, 3.3% of values were missing, and we performed listwise delete to exclude respondents who were missing data on any of the variables included (see flow diagram in Supplemental Fig. 1).

We used the limited dataset to examine cross-sectional bivariable relations between LCSEI scores and measures of global health as well as the covariates using weighted comparisons of means or proportions, as appropriate. We then estimated the independent association between LCSEI scores and mean GPH or GMH separately using multivariable linear regression adjusted for the covariates described above. The W5 all-waves regression weights were employed in our analysis, which accounted for oversampling at W1 and attrition between W1 and W5 in order to reduce bias and obtain estimates for the U.S. population.

### Sensitivity analysis

Many of the survey respondents with missing data who were excluded from the limited analytic dataset were missing responses on tobacco use variables of interest because they reported never smoking at W1 and current or former smoking at one of the later waves; variables with missing responses at W5 included age of smoking onset (n = 2,017), current smoking status (n = 905), and cigarette pack-years (n = 2,297) (not mutually exclusive). We therefore conducted a sensitivity analysis to determine whether results would change with the inclusion of these respondents. To do so, we first coded respondents who reported regular cigarette smoking at W2–5 but who were missing age of cigarette onset data from W1 as starting smoking at > 15 years of age. Then, we created an additional LCSEI category for respondents who were missing data on their current cigarette smoking status, age of cigarette onset, and/or cigarette pack-years at W5 (coded as 99). After recoding (which added a total N = 2,563 (82% of missing cases) back into the model), we ran a multivariable regression model with LCSEI as a categorical variable.

Tobacco-related diseases would be expected to mediate the association between lifetime cigarette smoking and health-related quality of life in older adults. Therefore, in a separate sensitivity analysis, we assessed how adding tobacco-related diseases to the multivariable model affected the association between the LCSEI and both global measures of health. We hypothesized that adding a measure of tobacco-related diseases would substantially reduce the association between the LCSEI and HRQOL outcomes. We used a disease comorbidity index that included conditions specific to tobacco use, which were COPD, diabetes, any cancer, congestive heart failure, high blood pressure, heart attack, osteoporosis, peripheral vascular disease, stroke, ulcer(s), high cholesterol, asthma, gum disease, pre-cancerous oral lesions, lost bone around teeth, stomach or gastrointestinal bleeding, cataracts or glaucoma, and macular degeneration. The comorbidity index ranged from 0–18; Cronbach’s alpha was 0.70; and test-retest reliability from wave-to-wave was 0.96.

## RESULTS

### Description of the Sample.

The sample (N=7,001) was largely representative of the US population over 40y (Table 1), with higher percentages of females (54.6%), with Black adults representing 11.3%, and with 24.5% having annual incomes of <$25,000. Regarding tobacco risk factors, 6.7% smoked during childhood, and 16.9% currently smoked (2.1% <10cig/d, 4.8% 11–20 cig/d, and 10.0% >20 cig/d). The weighted mean (std dev) for pack-years of smoking among adults who ever smoked was 32.9 (48.0), with 21.5% having >40 pack-years, and hours of exposure to second-hand smoke per week was 3.6 (13.4), with 4.6% reporting >20 hours/w. With respect to obesity, 38.1% had a body mass index of 30 or more. Mean (std dev) for GPH and GMH were 14.8 (3.1) and 14.5 (3.8) respectively. Table 2 shows the percentile distribution of each outcome variable.

Table 2 gives the sample distribution for the LCSEI, along with means for global physical and mental health at each of the index levels. Weighted mean for the LCSEI was 2.4 (std dev = 2.9) for this sample. Sample percents at each index level ranged from 41.5% (LCSEI = 0) to 0.7% (LCSEI = 11); LCSEI scores of 1 and 4 were the most prevalent nonzero levels (13.0% and 10.3% respectively), and 15.6% had a score of 5 or more.

Figure 1 shows the bivariable relation between scores on the LCSEI and mean GPH (Panel A) and GMH (Panel B). Both showed consistent negative linear associations (R^2^ = 0.95 for each plot); GPH and GMH declined by an average of −0.28 and −0.30 points respectively for each 1-point increase in the LCSEI.

### Multivariable associations

After adjusting for sociodemographics, weekly exercise, and obesity, average GPH and GMH declined by −0.20 (95% confidence interval= −0.23, −0.17) and −0.22 (−0.25, −0.18) points respectively for each 1-point increase in the LCSEI (Table 3). To better understand the magnitude of this effect size, we compared the size of the effect for the LCSEI on global health to effect sizes for sociodemographic covariates. The decrease in global health associated with a 1-point increase in LCSEI score was similar to the size of the decrease associated with a decade of aging (for GPH), female sex (for GMH), or moving one lower income category (for both GMH and GPH). Global physical and mental health was also negatively associated with obesity (−1.14 [−1.25, −0.94] and −0.54 [−0.75, −0.33] respectively) but not exercise. Age had a variable association with GMH, with the only significant comparison to the 40–49 referent category being 70–79, which had a significantly higher mean (0.34). Black adults had significantly better GMH scores compared to White adults and no difference in GPH scores after covariate adjustment.

**Figure 2:**
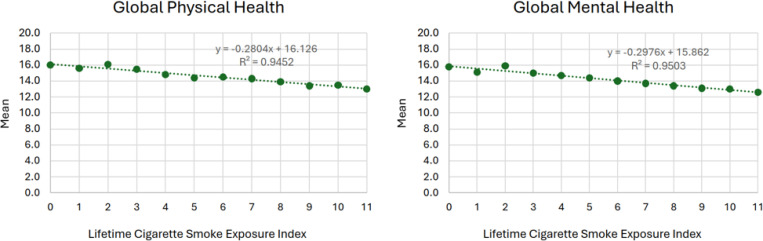
Figure 1. Bivariable association between lifetime cigarette smoke exposure index (LCSEI) scores and global physical and mental health.^1^ 1 Higher scores indicate a higher health-related quality of life

Figure 2 compares the multivariable effect sizes for the LCSEI, income, and education with GPH (Panel A) and GMH (Panel B). For the LCSEI, the figure shows the effect of a score of 11 (vs 0); for household income, the figure shows the effect of <25K/year (vs ≥100K/year); for education, the figure shows the effect of not finishing high school/GED (vs Bachelor’s/advanced degree). For both GPH and GMH, the strength of the association for the LCSEI (−2.14 [−1.52, −2.77]) and −2.16 [−1.43, −2,89] for GPH and GMH respectively) was significantly higher than for education (−0.44[−0.22, −0.67] and −0.77 [−0.38, −1.15]) and was about 30% higher than for household income (−1.61 [−1.27, −1.94] and −1.65 [−1.26, −2.04]).

### Sensitivity analysis

After entering respondents who were missing information on smoking history in multivariable regressions (N=2,563; 82% of missing cases), the associations between the LCSEI and GPH and GMH remained largely unchanged (Table 4). The estimates for respondents coded as 99 (missing data on the LCSEI) were similar to the reference category of respondents with LCSEI values of 0 both for GPH (−0.20 [−0.42, 0.03]) and GMH (−0.29 [−0.57, −0.01) and the inclusion of these respondents did not confound primary findings.

After addition of the comorbidity index to the multivariable regressions, the association between the LCSEI and GPH (−0.13 [−0.15, −0.10]) and GMH (−0.16 [−0.19, −0.12]) remained statistically significant for the subsample of respondents who had comorbidity data (N=6,255). GPH declined by an average of −0.43 (−0.48, −0.38) and GMH declined by an average of −0.34 (−0.39, −0.29) for each additional 1-point increase on the comorbidity index.

## DISCUSSION

We developed a comprehensive measure of lifetime cigarette smoke exposure (LCSEI), which allowed us to consider the combined effects of cigarette smoking status, heaviness of current cigarette smoking, pack-years of smoking, childhood smoking, and second-hand smoke exposure on HRQOL in older adults. The LCSEI showed a negative linear association with HRQOL, underlining the additive effect for each component of the LCSEI. Moreover, the full range of exposure was associated with a decline of about two-thirds of a standard deviation for global physical and mental health, a moderately large effect size. (21) The association between LCSEI scores and HRQOL was larger than any other single risk factor, greatly exceeding the association for education (< high school/GED vs advanced degree) and rivaling the influence of poverty compared to affluence (household income <$25K/year vs. ≥100K/year).

Many studies to date have examined the relation between cigarette smoking and HRQOL but few have included multiple dimensions of cigarette exposure. Drawing from and updating the review by Goldenberg et al,(2) we found fourteen studies(1, 3, 4, 7–10, 22–28) that matched ours in that they gave cross-sectional associations between individual cigarette use and HRQOL and studied samples that were population-based (not disease-based). Eight of these reported associations that were small in magnitude(3, 4, 9, 10, 23, 26–28) on the order of 0.2 standard deviation,(21) another five reported small-to medium size associations (some 0.5 or more standard deviation),(1, 7, 8, 24, 25) and we were unable to determine the strength in one.(22) The first set of studies had similarities that help explain the small association: five compared HRQOL associations for adults who currently smoke with those who formerly or never smoked (without regard to heaviness or duration),(3, 9, 10, 23, 27) five included young adults in the sample,(3, 9, 26–28) and four provided estimates that were adjusted for smoking-related diseases.(3, 9, 26–28) Of the five studies that reported some moderately large associations, four found moderately strong associations for those who smoked heavily,(1, 7, 8, 24) and one examined respiratory quality of life in a sample with high rates of lung disease.(25) None of the studies considered second-hand smoke exposure or age of onset of smoking.

Our approach to the question of cigarette smoking and HRQOL differs in that we wished to estimate the full impact of lifetime cigarette smoke exposure in older adults, those who have experienced many decades of exposure through active smoking and second-hand smoke. Moreover, this study considered the possibility that exposure to cigarette smoke during critical phases of development (adolescence) could have impacted HRQOL over-and-above duration of exposure. Finally, we carefully considered covariate adjustment in the multivariate model. Given that the relation between lifetime cigarette smoke exposure and smoking-related diseases is considered causal, we felt that it was inappropriate in a cross-sectional analysis to adjust for these diseases. Instead, we considered them mediators of the effects of lifetime cigarette smoking on HRQOL.

Adding tobacco-related disease covariates reduced the size of the association for LCSEI by about 50%, confirming the important mediating influence of these diseases on HRQOL. Consistent with some of the studies cited above,(3, 9, 26–28) we found small but significant associations between cigarette smoking and HRQOL after accounting for smoking-related diseases. This could mean that cigarette smoke exposure has concurrent influences on HRQOL over-and-above influences mediated by disease, or there could be influences on adolescent development that negatively affect adult perceptions of HRQOL, or incomplete measurement of the many diseases caused by smoking. More research is needed to help us understand the mechanisms through which lifelong exposure to cigarette smoke affects quality of life beyond effects mediated by the chronic diseases caused by smoking.

This study has strengths, some of which rest on the ability to assess lifetime exposure to cigarette smoke in the PATH Study, with many variables incorporated into the LCSEI that would not have been available to earlier investigators. The variables allowed us to develop an index that captures lifetime exposure to cigarette smoking and has good reliability and a linear association with HRQOL. The PATH Study is a longitudinal survey of a national US population sample, another strength. Use of weights allows us to address attrition bias from the baseline PATH Study interview. There are also weaknesses. The study is cross-sectional, which could be taken as a weakness because of the idea that perceptions of quality of life could bias recall of cigarette smoke exposure and bias the estimates. As another limitation, although missing item response was less than 5% on average, it resulted in a large loss of the sample in the primary analysis. However, we found that adding 82% of respondents with missing data back into the sample in a sensitivity analysis did not confound primary findings. Finally, there is the possibility that missing confounders could explain some of the impact we attribute to cigarette smoke exposure.

## CONCLUSIONS

Previous research on the impact of cigarette smoking on HRQOL may have underestimated the effects of smoking because of narrow measurement of the exposure, inclusion of young adults in the sample, and adjustment for causal disease mediators of the association. Our research suggests that lifetime exposure to cigarette smoke has moderately large influences on physical and mental quality of life, as large as the impact of socioeconomic indicators like education and income. More research is needed to better understand how cigarette smoking affects HRQOL over-and-above smoking-related disease, and to determine whether these findings apply across countries at different income levels.

## Supplementary Material

Supplement 1

## Figures and Tables

**Figure 3:**
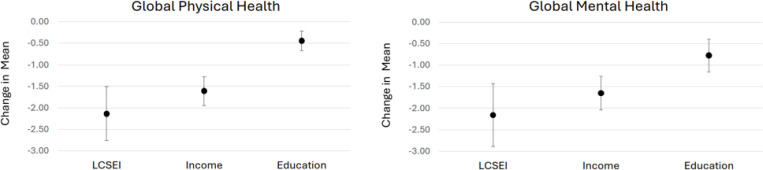
Figure 2. Adjusted decreases in mean for global physical and mental health for high (score of 11) vs low lifetime cigarette smoking (LCSEI),^1^ low vs high income,^2^ and minimal vs advanced education.^3^ 1 Lifetime cigarette smoke exposure index (LCSEI) 11 vs. 0. 2 Household income of <$25,000/yr vs. ≥ $100,000/yr. 3 Did not finish high school/GED vs. Bachelors or advanced degree.

## Data Availability

data are publicly available data from the Population Assessment of Health Study, available at https://www.icpsr.umich.edu/web/NAHDAP/studies/36231/datadocumentation

## Abbreviations

LCSEI: lifetime cigarette smoke exposure index
HRQOL: health-related quality of life
GPH: global physical health
GMH: global mental health
PATH: Population Assessment of Tobacco and Health
COPD: chronic obstructive pulmonary disease
PROMIS: Patient-Reported Outcomes Measurement Information System
W: Wave
BMI: body mass index
