# Supplementary material for: Comprehensive Assessment of Lifetime Cigarette Smoking and Its Association with Health-Related Quality of Life among Older US Adults—A Cross-sectional Study
Source: Res Sq. 2024 Nov 17:rs.3.rs-5319716. Preprint. [Version 1] doi: 10.21203/rs.3.rs-5319716/v1 (PMC11601830; doi:10.21203/rs.3.rs-5319716/v1)
Supplement: Supplement 1 [file NIHPPRS5319716V1-supplement-1.pdf]

Supplementary Files

This is a list of supplementary files associated with this preprint. Click to download.

- Tables.docx
